# Computed tomography-based deep-learning prediction of lymph node metastasis risk in locally advanced gastric cancer

**DOI:** 10.3389/fonc.2022.969707

**Published:** 2022-09-23

**Authors:** An-qi Zhang, Hui-ping Zhao, Fei Li, Pan Liang, Jian-bo Gao, Ming Cheng

**Affiliations:** ^1^ Department of Radiology, The First Affiliated Hospital of Zhengzhou University, Zhengzhou, China; ^2^ Department of Radiology, Shaanxi Provincial People’s Hospital, Xi’an, China; ^3^ School of Cyber Science and Engineering, Wuhan University, Wuhan, China; ^4^ Department of Medical Information, The First Affiliated Hospital of Zhengzhou University, Zhengzhou, China; ^5^ Henan Key Laboratory of Image Diagnosis and Treatment for Digestive System Tumor, The First Affiliated Hospital of Zhengzhou University, Zhengzhou, China

**Keywords:** deep learning, locally advanced gastric cancer, lymph node metastasis, radiomics, computed tomography

## Abstract

**Purpose:**

Preoperative evaluation of lymph node metastasis (LNM) is the basis of personalized treatment of locally advanced gastric cancer (LAGC). We aim to develop and evaluate CT-based model using deep learning features to preoperatively predict LNM in LAGC.

**Methods:**

A combined size of 523 patients who had pathologically confirmed LAGC were retrospectively collected between August 2012 and July 2019 from our hospital. Five pre-trained convolutional neural networks were exploited to extract deep learning features from pretreatment CT images. And the support vector machine (SVM) was employed as the classifier. We assessed the performance using the area under the receiver operating characteristics curve (AUC) and selected an optimal model, which was compared with a radiomics model developed from the training cohort. A clinical model was built with clinical factors only for baseline comparison.

**Results:**

The optimal model with features extracted from ResNet yielded better performance with AUC of 0.796 [95% confidence interval (95% CI), 0.715-0.865] and accuracy of 75.2% (95% CI, 67.2%-81.5%) in the testing cohort, compared with 0.704 (0.625-0.783) and 61.8% (54.5%-69.9%) for the radiomics model. The predictive performance of all the radiological models were significantly better than the clinical model.

**Conclusion:**

The novel and noninvasive deep learning approach could provide efficient and accurate prediction of lymph node metastasis in LAGC, and benefit clinical decision making of therapeutic strategy.

## Introduction

Gastric cancer (GC) is one of the most common cancers and the third leading cause of death from cancer worldwide (1). The incidence rate of gastric cancer is relatively high in Asia, South American and Europe (2–4). Locally advanced gastric cancer refers to the wall invasion deeper than the submucosa, with a high rate of lymph node metastasis (LNM) and poor clinical prognosis (5–7). Accurate evaluation on lymphatic metastasis based on preoperative computed tomography (CT) images is crucial for individual treatment of LAGC (8–10). Preoperative knowledges of LNM have important clinical significance for selecting the optimal surgical procedure (endoscopic procedures or gastrectomy plus lymph node dissection) and the need for adjuvant therapy (11–13). The National Comprehensive Cancer Network recommended CT as a first-line imaging technique for detecting LNM, but the overall accuracy is 50%-70%, which is unsatisfactory (14).

The advances in deep learning techniques provides a new field for CT imaging analysis, which could convert medical images to mineable data and generate thousands of quantitative features (15). Convolutional neural networks (CNNs) have been proved to be an effective method for improving the diagnostic accuracy of medical imaging (16–18). Due to the lack of enough annotated cases, training a CNN model from scratch for one specific clinical problem often is infeasible. An effective approach is to adopt the transfer learning technique using pre-training CNNs, which ran additional steps of pre-training on specific medical domain from the existing checkpoint. It is frequently used to alleviate the limitations of small datasets and expensive annotation (19, 20). Part of natural imaging descriptors developed for object detection have been used for lesion segmentation in medical imaging analysis (21). Another option is to use a pretrained CNNs models as the feature extractor and traditional machine learning methods as classifier, which may also have satisfactory performance in terms of prediction accuracy and computational cost (22). Handcrafted radiomics have been studied extensively for radiological diagnosis and prediction (8, 23, 24). However, the application of transfer learning to prediction of LNM in gastric cancer has not been explored.

In this study, we hypothesize that CT-based transfer learning techniques are feasible to extract deep learning features for preoperatively predicting LNM risk. To this end, our study aims to build a noninvasive measurement based on pre-trained deep learning models for the preoperatively prediction of LNM in patients with gastric cancer, making comparison with the handcrafted radiomics method. Additionally, we further explored the application value of deep learning features in predicting LNM and making treatment decisions.

## Materials and methods

### Patients

**Figure 1:**
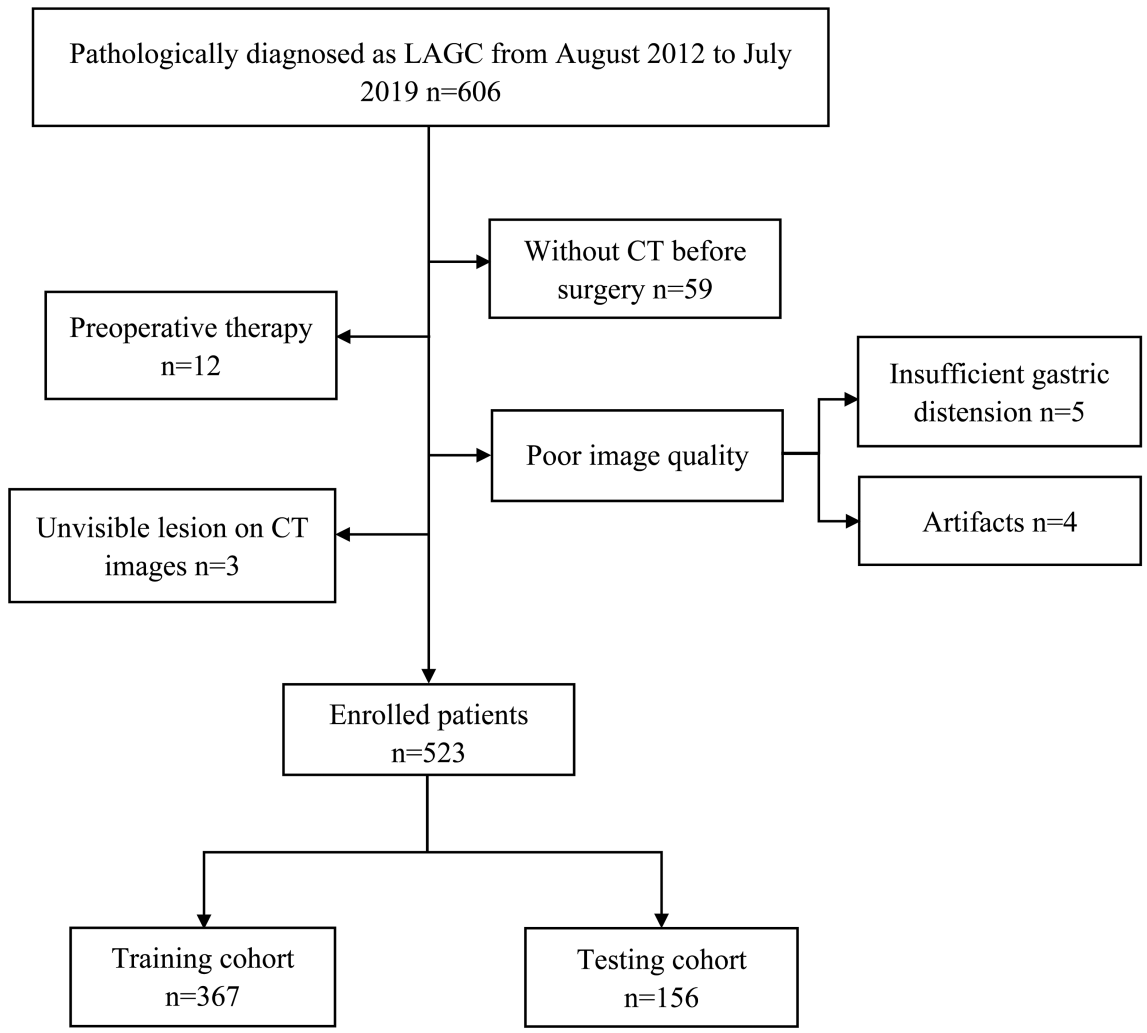
Flow chart of patient selection.

This retrospective study was approved by the institutional review board of our hospital, and the requirement for informed consent was waived. A total of 523 consecutive patients with gastric cancer who was treated between August 2012 and July 2019 were enrolled. The patients were enrolled based on following inclusion criteria: (a) pathologically diagnosed as local advance gastric cancer (pT2-4aNxM0); (b) all patients with gastrectomy plus lymph node dissection and CT imaging data were complete; (c) without any systematic or local treatment before CT imaging study or surgery; (d) the lesion covers at least 3 slices on CT cross section. The patients were excluded based on the following criteria: (a) invisible lesion on CT images; (b) insufficient stomach distension; (c) poor image quality for post-processing due to artifacts. The flowchart of patient selection was shown in **Figure 1**. We adopted computer-generated random numbers to split the training cohort (n=367, 74.40% males; mean age, 59.75 ± 10.38; range, 22-82 years) and the testing cohort (n=156, 73.98% males; mean age, 59.36 ± 9.94; range, 22-81 years). The tumor location information was got from the medical or endoscopic reports, and the clinical information was got by reviewing the medical reports.

### Image process and tumor segmentation

All patients underwent contrast-enhanced CT scan and informed consent forms were signed before inspection. The CT scans were acquired with breath-hold with the patient head first supine in all of the phases for covering the whole abdomen. The details on CT acquisition parameters were described in **Supplemental Material**.

Tumor regions of interest (ROIs) were manually segmented CT images by two experienced radiologists using ITK-SNAP software (version 3.6.0; http://www.itksnap.org). In order to make a fair comparison with different features, we only chosen one slice with the maximum cross-sectional area of the tumor lesion by the radiologists. We randomly chosen 30 patients from training cohorts to assess the interobserver reproducibility for ROI-based radiomics features in a blinded manner. After one month, segmentation procedure was repeated to assess the intraobserver reproducibility. The features with intra-class correlation coefficient (ICC) greater than 0.75 were selected for further analysis. For deep learning features extraction, the 3 axial slices containing the delineated tumor were resized to 224× 224mm (the size for the input layer of the pretrained CNN models) with the use of a bounding box covering the radiologist contoured tumor area.

### Deep learning features

We employed five commonly convolutional neural networks (ResNet (25), VGG16 (26), VGG19 (26), Xception (27) and InceptionV3 (28)) as base models to extract deep learning features automatically. These five CNNs models were pre-trained on the large-scale lightweight well-annotated biomedical image database (29). We removed the last fully connected layer at top of the network, and applied global max pooling strategies to efficiently capture the maximum values of each layer of the feature maps. Finally, we converted the feature maps to the raw values. The extracted deep features were used to construct the machine learning model. Due to the complexity of deep learning model structure, the potential mechanisms of predictive value were unclear. Additional details of deep features extraction in this study are listed in **Supplementary Material**. Furthermore, Gradient-weighted Class Activation Mapping technique (Grad-CAM) could generate visual explanations for any CNN-based model (30). We use this visualization technique to investigate which regions of the ROI were most important in the deep features.

### Radiomics features

Image standardization was implemented before feature extraction: bi-cubic spline interpolation was used to resample the image scale in the slice to reduce the heterogeneity results from different scanners, resulting in a voxel size of 1mm×1mm×1mm (31, 32). The radiomics features were automatically extracted from each radiologist’s ROIs using the Python package Pyradiomics (http://pyradiomics.readthedocs.io) (33). The radiomics features were standardized by referring to the Image Biomarker Standardization Initiative (IBSI) (34). The study was based on the reporting guidelines of IBSI. The hand-crafted radiomics features were divided into three different groups of features: shape features, histogram statistics, second order features: Gray Level Co-occurrence Matrix (GLCM), Gray Level Dependence Matrix (GLDM), Gray Level Run Length Matrix (GLRLM), Gray Level Size Zone Matrix (GLSZM), Neighboring Gray Tone Difference Matrix (NGTDM). Most features mentioned above were delineated according to the IBSI, and the detailed introduction of the features were described in **Supplementary Material**.

### Harmonization

Radiomics extracts features from medical images more precisely than general visual evaluation. However, radiomics features are affected by the acquisition protocol and reconstruction methods, thus obscuring underlying biologically important texture features. In practical clinical retrospective studies, it is impractical to standardize the parameters of different devices in advance. In order to reduce the batch effect, ComBat harmonization technique had been successfully applied to properly correct radiomic feature values from different scanner or protocol effect (35). We exploited the ComBat to pool and harmonize radiomics and deep learning features after extraction.

### Feature selection and model construction

Based on the training set, we performed deep learning or radiomics feature selection and constructed model for predicting lymph node metastasis. Firstly, the z-score normalization was used for standardization. In addition, we selected top 20% best features by univariate analysis. Then, we used an embedded feature selection approach based on the least absolute shrinkage and selection operator (LASSO) algorithm to select the most predictive features. Classification model was constructed by the SVM (36). We also built a clinical model based on the clinical characteristics. The code for model construction is available on Github (https://github.com/cmingwhu/DL-LNM).

### Statistical analysis

P values for differences in the clinical characteristics between cohorts were assessed by Fisher’s exact test or Chi-square test for categorical variables, and the Mann-Whitney U test or independent t-test for numeric variables. Receiver operating characteristic curve (ROC) was adopted to determine the predictive performance of the related models, while the DeLong’s test was adopted for comparison of AUC between each model. The AUC and 95% confidence interval (CI) were calculated. Accuracy, specificity and sensitivity were calculated to assess the diagnostic performance. The calibration of the model was evaluated by the calibration curves using the Hosmer-Lemeshow test. To assess the reproducibility of our results, we randomly divided the patients into training or testing set ten times. Subsequently, the model was reconstructed and validated repeatedly. P value < 0.05 was considered statistically significant. We used Python version 3.6 (https://www.python.org/) and R version 4.0.3 (https://www.r-project.org) to perform statistical analysis and graphic production. The packages used in this study are shown in **Supplementary Material**.

## Results

### Clinical characteristics

**Figure 2:**
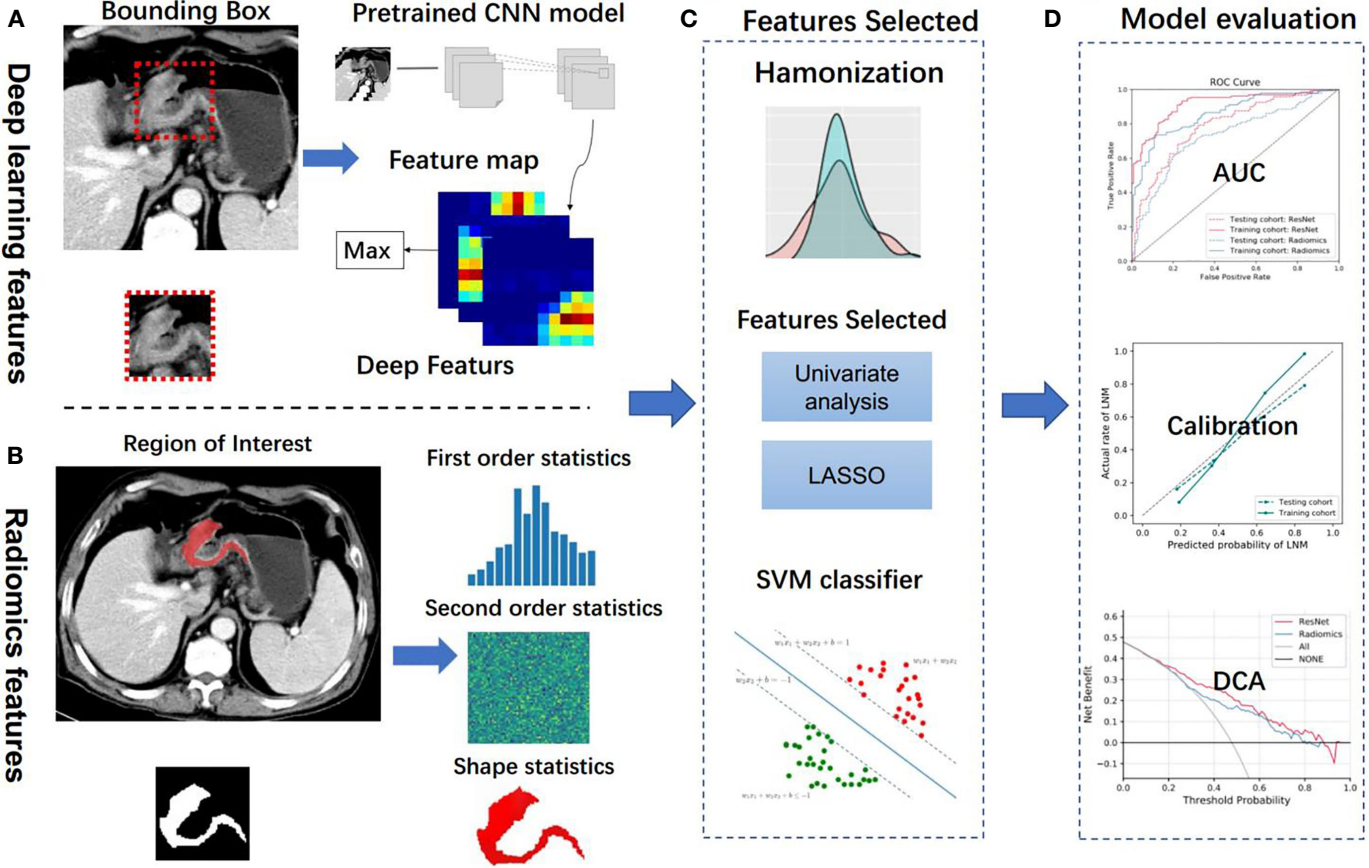
Analysis flowchart. **(A, B)** Features extraction from the deep learning method and handcrafted radiomics method. **(C)** Machine learning methods were employed in model construction. **(D)** Model evaluation. CNN, convolutional neural network; LASSO, the least absolute shrinkage and selection operator; SVM, support vector machine; AUC, area under the receiver operating characteristic curve; DCA, decision curve analysis.

**Figure 2** depicts the workflow processes. Of the 523 patients (mean age: 59.64 ± 10.24 years; male: 74.40%) with locally advance gastric cancer for this study, 367 patients were assigned to the training cohort, and 156 patients was assigned for testing cohort. Clinical characteristics in two cohorts are shown in **Table 1**. No significant difference was identified in terms of sex, age, tumor location, tumor thickness between the two cohorts (**Tables 1**, **S1**). Tumor diameter, clinical T stage, and CT-reported LN differed significantly between LNM-negative and positive group in two cohorts (p <0.05). Finally, a clinical model was established (incorporating tumor diameter, CT-reported LN and clinical T stage) for predicting LNM, yielding an AUC of 0.683 and 0.756 for testing and training cohorts, respectively, as shown in **Tables 2**, **S2**.

**Table 1:** The clinical characteristics of patients in the training and testing cohorts.

Characteristics	Training cohort (120: 247)			Testing cohort (47: 109)		
	LNM (-)	LNM (+)	P value	LNM (-)	LNM (+)	P value
Age (mean ± SD, years)	59.98 ± 10.53	59.83 ± 10.40	0.834	60.98 ± 10.91	58.66 ± 9.46	0.182
Sex						
Female	28 (23.3)	66 (26.7)	0.569	16 (13.04)	24 (22.0)	0.168
Male	92 (76.7)	181 (73.3)		31 (86.96)	85 (78.0)	
Location						
Cardia/fundus	67	130	0.453	26	52	0.655
Body	23	51		11	26	
Antrum	29	57		10	29	
More than two-thirds of stomach	1	9		0	2	
Tumor thickness ± SD (mm)	22.65 ± 8.58	23.43 ± 7.70	0.383	21.71 ± 7.67	21.93 ± 7.46	0.865
Tumor diameter ± SD (mm)	82.60 ± 41.59	94.22 ± 51.70	0.032*	70.29 ± 30.46	90.36 ± 51.69	0.014*
Clinical T stage						
T2	13	21	0.005*	9	13	0.006*
T3	81	130		33	57	
T4a	26	96		5	39	
CT-reported LN						
Negative	90	76	<0.001*	39	37	<0.001*
Positive	30	181		8	72	

LNM, lymph node metastasis; (-), negative; (+), positive; *p < 0.05.

**Table 2:** Predictive performance of radiological or clinical models in the testing cohort.

	AUC	Accuracy	Sensitivity	Specificity	PPV	NPV
InceptionResNetV2	0. 707	65.6	67.9	60.8	78.3	47.7
	(0.653, 0.771)	(60.1, 72.7)	(55.9, 72.2)	(56.2, 73.3)	(73.0, 85.3)	(31.7, 53.8)
VGG16	0.661	61.8	65.2	58.0	63.2	60.0
	(0.540, 0.745)	(55.7, 70.6)	(60.6, 69.9)	(51.8, 65.8)	(55.5, 70.0)	(53.7, 69.4)
VGG19	0.578	49.6	40.6	68.6	72.9	35.7
	(0.507, 0.661)	(41.7, 55.1)	(40.6, 51.9)	(63.0, 75.6)	(66.0, 80.6)	(30.6, 47.9)
ResNet50	0.796	75.2	80.2	64.7	82.5	61.1
	(0.715-0.865)	(67.2, 81.5)	(75.4, 84.2)	(58.2, 71.6)	(74.9, 87.3)	(55.5, 69.3)
Xception	0.660	62.4	65.1	56.9	75.8	43.9
	(0.607, 0.759)	(56.2, 71.6)	(52.2, 69.0)	(49.8, 68.7)	(70.9, 81.1)	(40.9, 51.6)
Radiomics	0.704	61.8	56.5	73.5	82.4	43.4
	(0.625, 0.783)	(54.5, 69.9)	(50.8,62.3)	(68.8, 79.8)	(75.8,87.3)	(40.8, 52.1)
Clinical signature	0.683	68.2	67.6	67.7	70.7	53.6
	(0.632, 0.721)	(65.3, 72.1)	(63.5,70.1)	(63.1, 71.6)	(67.5, 75.2)	(50.7, 61.9)

### Handcrafted radiomics model construction

851 handcrafted radiomics features were extracted, where 107 were from the original images and 744 were from the wavelet filtered images. After ComBat harmonization (35). Forty-eight features were selected, including three and forty-five from original and wavelet filtered images (**Table S3**). The handcrafted radiomics model get an AUC of 0.704, C-index of 0.704, accuracy of 61.8%, sensitivity of 56.5%, specificity of 73.5%, positive predictive value (PPV) of 82.4%, and negative predictive value (NPV) of 43.4% in the testing cohort, and an AUC of 0.779, C-index of 0.779, accuracy of 74.0%, sensitivity of 77.5%, specificity of 66.4%, positive predictive value (PPV) of 83.2%, and negative predictive value (NPV) of 57.9% in the training cohort in **Tables 2**, **S2**.

### Deep learning model construction

**Figure 3:**
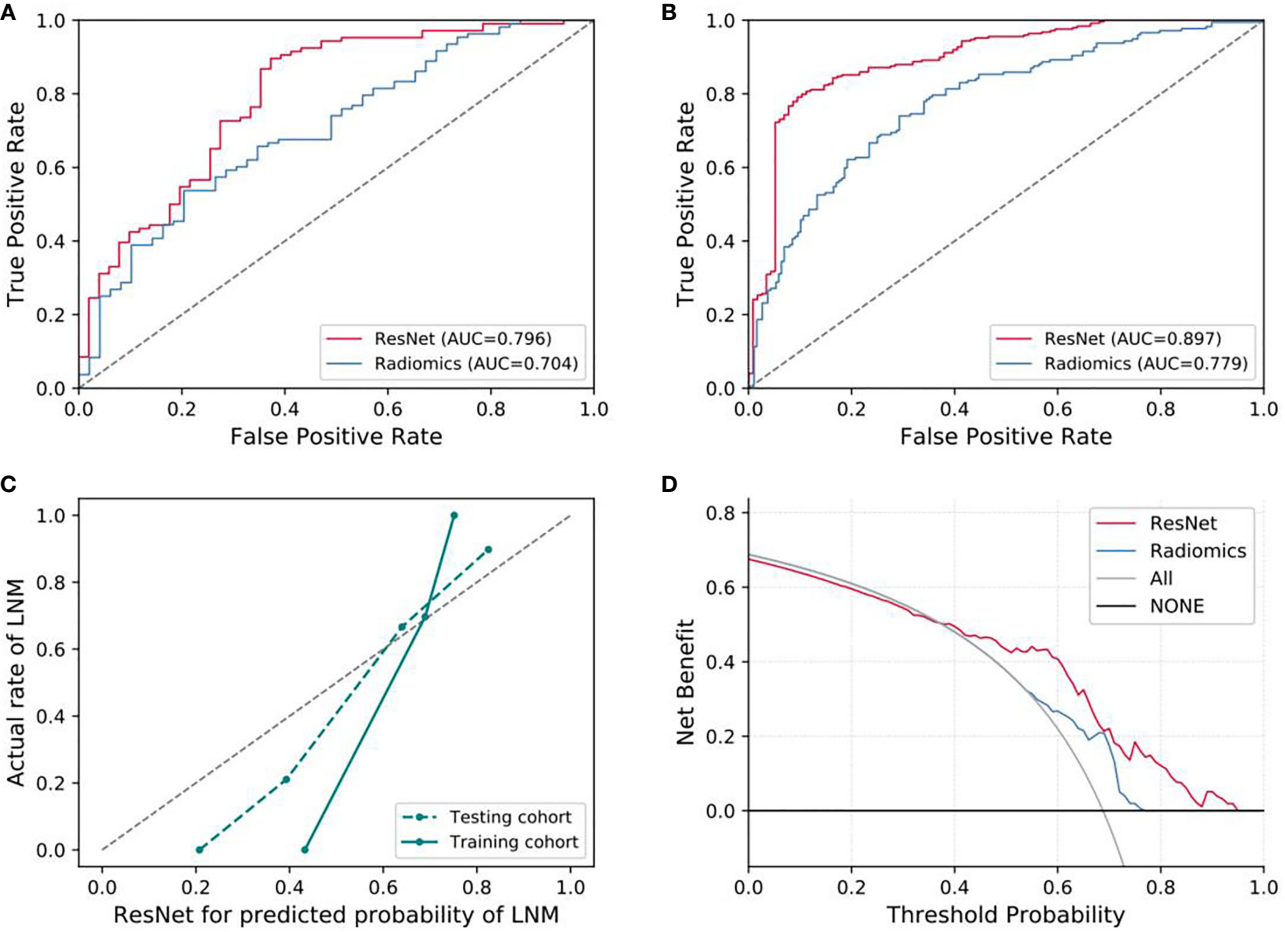
Evaluation of predictive performances for ResNet-SVM model and radiomics model. **(A)** The ROC curves showing the predictive performances of the ResNet and the radiomics model in testing cohorts. **(B)** The ROC curves showing the predictive performances of the ResNet and the radiomics model in training cohorts. **(C, D)** Curves of calibration analysis and the decision curve analysis for the ResNet and radiomics model. AUC, area under the receiver operating characteristic curve; LNM, lymph node metastasis.

**Figure 4:**
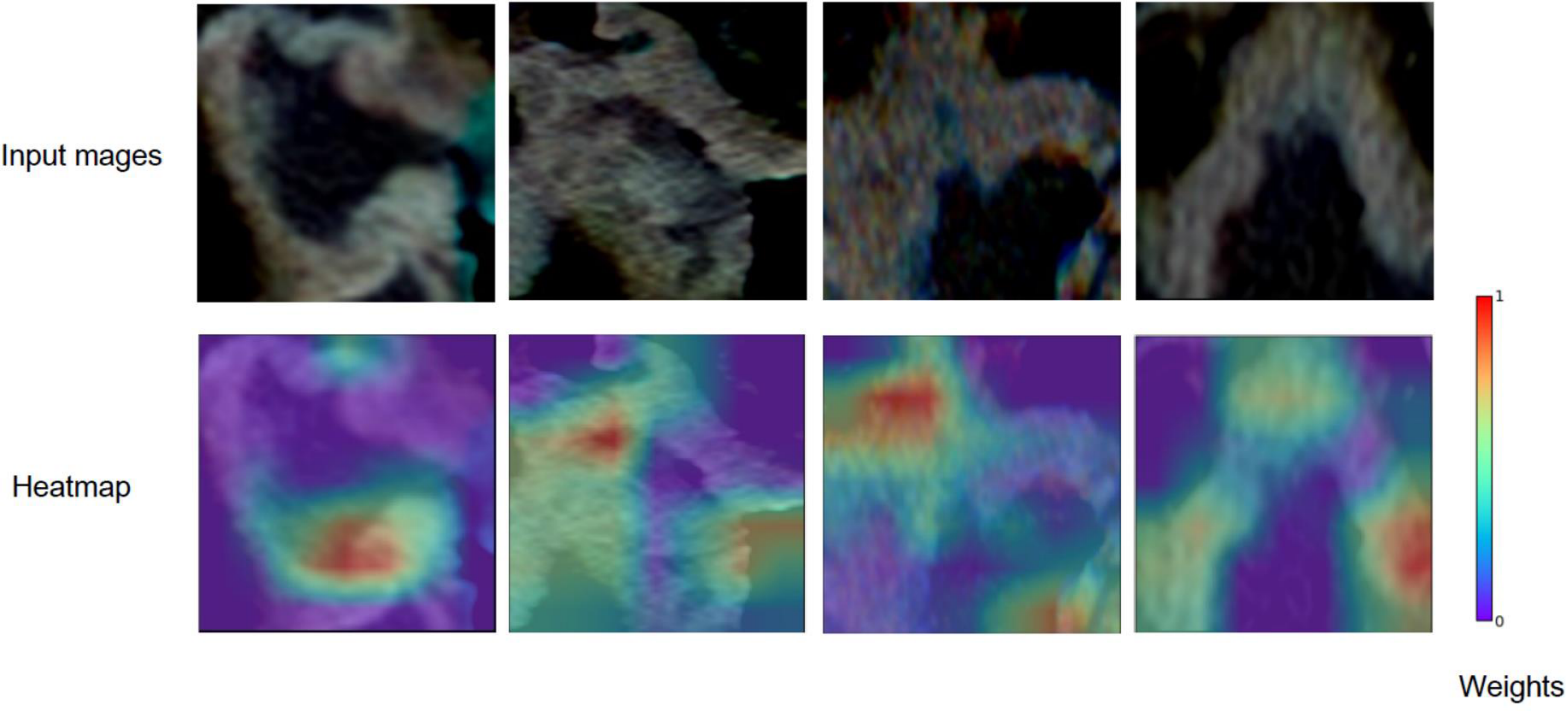
Grad-CAM visualizations for the feature heatmaps of representative patients generated from the ResNet. The right color bar indicates the scaled weights of deep features.

For predicting LNM based on deep learning features, we compared five CNNs models which were adopted to extract deep features to optimize the prediction performance. The AUC ranged from 0.578 to 0.796 for testing cohort, and 0.804 to 0.897 for training cohort, as shown in **Table 2**, **S2**. The ResNet-SVM model containing 116 deep learning features could get the best classification performance among the five CNNs models and was superior to the radiomics model, and yielding an AUC of 0.796, C-index of 0.796, accuracy of 75.2%, sensitivity of 80.2%, specificity of 64.7%, PPV of 82.5%, NPV of 61.1% in the testing cohort in **Figures 3A, B**. The calibration and favorable clinical benefit could also get good performance in **Figures 3C, D**. The number of features were adopted in model of different CNNs are listed in **Table S4**. Features maps from the ResNet model could indicate the locations that were important in generating the output. With the segmentation of the tumor region delineated, the informative slices (one slice with the maximum tumor area) were cropped to 224 × 224 mm using a bounding box covering the whole tumor area. The cropped images were used to generate the features from ResNet and the visualization of feature heatmaps were generated based on the Guided Grad-CAM, as shown in **Figure 4**. The tumoral lesion and perifocal areas in images were of great valuable for the feature pattern extraction. Then, we further analyzed the performance generated by features extracted from different layers to see whether the last layer was the most suitable to extract features. The current features extraction strategy is the best for ResNet in **Table S5**.

Different classifiers and features selection methods could greatly affect the prediction performance. For the features extracted from different CNNs, we compared the cross combination of multiple classifiers and feature selection methods. We find that the performances of different combinations are different, the results shown that the current combination method of classifier and extraction (ResNet-SVM) demonstrated the best discrimination ability with an AUC of 0.796 (95% CI, 0.715-0.865) for our dataset, as shown in **Figure S1** and **Table S6**, but further generalization tests on other datasets are required. The DeLong test showed that there were significant improvements in contrast to the radiomics model and the clinical signature (p < 0.05), which yielded AUCs of 0.704 (95% CI, 0.625–0.783) and 0.683 (95% CI, 0.632–0.721), respectively.

### Radiomics-deep learning combined model

We further integrated the radiomics and deep learning features to explore whether the predictive capability could be improved. After combination with radiomics and deep features, the prediction performance had not been improved, with a comparable AUC of 0.787 in the testing cohort in **Figure S2**. In addition, we further evaluated the addition of clinical factors to radiomics or deep learning features for potential improvement of prediction performance. The combination of deep and/or radiomics features with clinical features were incorporated into the model construction, the experimental results showed that combination of clinical factors could not increase the prediction performance in the testing cohort in **Figures S3, S4**.

## Discussion

In this retrospective study, we applied deep transfer learning techniques to build a CT imaging-based prediction model for LNM prediction in gastric cancer. Our previous studies shown that the noninvasive deep learning CT image-based radiomics model was effective for LNM prediction and prognosis in GC (37). Hereby, we adopted transfer learning technique and extract deep learning features from five different pre-trained CNNs. Finally, the ResNet-SVM model could achieve better performance than the handcrafted radiomics and clinical models. In addition, different gastric cancers have different potentials for lymph node metastasis due to the heterogeneity and complexity of primary tumors. Previous studies clarified that the tumor size were independent risk factors for LNM. Our results are consistent with the above studies, and it is reasonable that GCs with greater tumor size tend to have a higher risk of lymph node metastasis.

As an emerging image quantification approach, radiomics has been widely used in diagnosis and prognosis of cancer patients based on medical images (5, 38, 39). Previous studies mainly focused on the characteristic manifestations of CT imaging to develop radiomic model, and did not use the transfer learning technology in the field of radiological prediction of LNM in gastric cancer. We established a CT-based model using the novel deep learning technique. Deep learning features extraction only needs to set a fixed size bounding box to tumor area, which not only improves the efficiency, but also reduces the subjectivity of manual segmentation in the radiomics procedure.

Deep learning technology has been widely used in the field of medical image processing. However, training a deep learning model from scratch is often not feasible because of various reasons: (1) the lack of a number of annotated images for one specific clinical problem. (2) reaching convergence could take too long for experiments to be worth. In the medical domain, using pre-trained CNNs as feature extractors is an effective way to alleviate these issues (19, 39–41). Transfer learning can transfer prior knowledge of image features and apply it to medical imaging with better generalization and ease of replication and testing. Our research shows that deep learning features extracted by transfer learning approach generalized well in medical tasks and achieved fairly good results. Moreover, the combination of radiomics and deep learning features did not improve the prediction performance in our study (**Figures S2**), which is similar to the results published by (40, 41). The reason is that the imaging features calculated from different frameworks might have different high-level dimensional characteristics, which are not suitable for feature combination.

Our study has some limitations that are worth noting. First, tumor regions of interest were manually delineated on CT images, which is high cost and laborious task. Semi-automatic or automatic segmentation method may be better. Second, although our experimental results showed good prediction performance, indicating that transfer learning could alleviate the domain difference, heterogeneity existed between various dataset. The main obstacle of this research is the lack of sufficient annotated medical images to further train the deep learning models. Such dataset could further extract more valuable features to improvement prediction performance. Third, we followed the IBSI benchmarks to filter the images after resampling, which may lead to the failure of estimating how much this would affect wavelet features to some extent. Last, this study is a single-center which is lack of external validation for the developed model, but we further randomly divided the patients into training or testing set and reconstructed and tested repeatedly ten times to evaluate the results. And, we are working to further access our model in a bigger dataset that may come from multiple centers.

## Conclusion

In conclusion, our study adopted a noninvasive deep learning technique to perform prediction of LNM in GC. Compared to the handcrafted radiomics methods, the ResNet-SVM model could get better performance, and the implementation is simple and efficient without drawing the tumor contour manually. This study represented that the transfer learning strategy might also achieve good performance in medical imaging tasks without sufficient annotated medical images.

## Data availability statement

The original contributions presented in the study are included in the article/**Supplementary Material**. Further inquiries can be directed to the corresponding author. Requests to access these datasets should be directed to fccchengm@zzu.edu.cn.

## Author contributions

H-PZ and MC: design the research. A-QZ: performed the research. A-QZ and H-PZ: collected the data. FL and PL: analyzed the data. A-QZ and MC: analyzed the data and wrote the paper. J-BG and MC: reviewed the paper. All authors contributed to the article and approved the submitted version.

## Funding

This work was supported by the Key Project of Science and Technology Research of Henan Province (No. 222102210112), the National Natural and Science Fund of China (No. 61802350, 81971615), National Key Research and Development Program of China (No. 2019YFC0118803).

## Acknowledgments

This is a short text to acknowledge the contributions of specific colleagues, institutions, or agencies that aided the efforts of the authors.

## Conflict of interest

The authors declare that the research was conducted in the absence of any commercial or financial relationships that could be construed as a potential conflict of interest.

## Publisher’s note

All claims expressed in this article are solely those of the authors and do not necessarily represent those of their affiliated organizations, or those of the publisher, the editors and the reviewers. Any product that may be evaluated in this article, or claim that may be made by its manufacturer, is not guaranteed or endorsed by the publisher.
