# Supplementary material for: Computed tomography-based deep-learning prediction of lymph node metastasis risk in locally advanced gastric cancer
Source: Front Oncol. 2022 Sep 23;12:969707. doi: 10.3389/fonc.2022.969707 (PMC9537615; doi:10.3389/fonc.2022.969707)
Supplement: Supplementary file 1 [file DataSheet_1.docx]

Supplementary Material

# CT data acquisition

The CT scans were acquired with a 64-row CT scanner (Discovery CT or Revolution CT, GE Healthcare, Waukesha, WI, United States) or a 256-slice CT scanner (Brilliance iCT, Phillips Medical System, Netherlands). Conventional axial scanning was performed before and after an intravenous (i.v.) injection of nonionic contrast agent (Omnipaque, 350 mg I/ml, GE Healthcare, USA, 1.5 mL/kg and 3 mL/s) through a dual-head pump injector (Medrad, Warrendale, PA, United States). The scanning parameters were as follows: tube voltage, 120 kV; automatic mA technology is used for tube current; field of view (FOV), 500 mm; matrix, 512 × 512 mm; slice thickness, 0.625 mm to 5 mm; scan spacing, 0.625 mm to 5 mm. Finally, a 20-mL saline flush was performed at a rate of 3 mL/s. Arterial phase (AP) and venous phase (VP) CT images were collected after post-injection delays of 30 and 70 seconds, respectively. VP images in the DICOM format were retrieved for tumor segmentation because VP was the optimal phase for visualization of GC, and the performance of features extracted from VP images was slightly better than that of features extracted from AP images [1-3].

# Deep learning Feature definitions

**2.1 Data preprocessing**

With the segmentation of the tumor region delineated, the informative slices (one axial slices with maximum tumor area) were cropped to 224 mm * 224 mm (the size for the input layer of the used models) using a bounding box covering the whole tumor area. The cropped images with one axial slices as image channels were used as input of the convolutional neural network (CNN) model.

**2.2 CNN architecture**

In our study, VGG16, VGG19, ResNet, InceptionV3 and Xception [4-8] were used for the extraction of representational deep learning features. These networks were pretrained on ImageNet [9]. This publicly released dataset contains a large number of object categories and manually annotated training images. The optimization hyperparameters was not tuned, which meant a broader generalization on other datasets. The models are publicly assessible using Keras and TensorFlow open-source code (https://github.com/fchollet/deep-learning-models/releases/download/), under the MIT license. After preprocessing, one slices in CT images with the maximum area of the tumor lesion was propagated in the network to generate deep learning features.

**2.3 Removal of the last fully-connected layer**

For the pretrained models, the convolutional base is connected by a fully-connected layer. We removed the last fully connected layer. A total of 2048 (ResNet, InceptionV3 and Xception), 512 (VGG16, VGG19) feature maps were achieved from the new output of this model.

**2.4 Addition of max pooling layer and feature extraction**

With the use of a global pooling window, local data is concentrated to a decreased dimensionality.. After Step 2.3, for models with more than one dimensional features, we got feature maps with height and width dimensions, which corresponded to location invariance in the input layer. After global pooling, each feature map vector was transformed to a maximal raw value among them. The feature maps were transformed to numeric values, which were the representational deep learning features.

# Handcrafted radiomics Feature definitions

Handcrafted radiomics features were computed from the radiologist-drawn ROIs using an open-source python package PyRadiomics [10]. Detailed calculations of handcrafted radiomics features are described and provided in online documentation of PyRadiomics (https://pyradiomics.readthedocs.io/en/latest/features.html). The resampled voxel sizes were set to 1× 1 × 5 mm³ voxels to standardize the slice thickness. Image intensities were binned by 25 HU and voxel array shift were set on 1000. Segmented voxels were resampled at the range of 50 to 400 HU including the whole tumor and excluding the air and bone tissues. Defined radiomic image features without/after wavelet filtration that described tumor characteristics were extracted. Wavelet filtration filtered original image directionally with x, y and z directions respectively (H: High pass filter, L: Low pass filter), yielding 8 different combinations of decompositions. The extracted radiomics features can be divided into 3 groups: (I) first-order statistics, (II) shape features, and (III) second-order features. Most features defined below were in accord with feature definitions as described by the Imaging Biomarker Standardization Initiative (IBSI), which were available in a separate document by Zwanenburg et al.[11]. There are differences in gray value discretization (for the fixed bin size type) and resampling that cannot be corrected by customization settings alone and require replacement by custom functions, which are elaborated in the Pyradiomics documents. It is worth noting that two features available in PyRadiomics are not defined in the IBSI, namely: Total Energy and Standard Deviation. Entropy in Pyradiomics is defined by IBSI as Intensity Histogram Entropy. Uniformity in Pyradiomics is defined by IBSI as Intensity Histogram Uniformity. Mesh Volume in Pyradiomics is defined as Volume. Voxel Volume in Pyradiomics is defined in IBSI as Approximate Volume. Joint Energy in Pyradiomics is defined by IBSI as Angular Second Moment. Maximum Probability in Pyradiomics is defined by IBSI as Joint maximum Sum of Squares in Pyradiomics is defined by IBSI as Joint Variance. The PyRadiomics kurtosis is not corrected, where IBSI kurtosis is corrected by -3, yielding 0 for normal distributions. All the remaining features correspond to the definitions provided by IBSI.

Here, we describe the radiomic features with definitions or implementation that differ from the IBSI reference document used in our model.

**3.1 First Order Features**

First-order statistics describe the distribution of voxel intensities within the image region defined by the mask through commonly used and basic metrics.

Let:

- 𝐗 be a set of 𝑁𝑝 voxels included in the ROI
- 𝐏(𝑖) be the first order histogram with 𝑁𝑔 discrete intensity levels, where 𝑁𝑔 is the number of non-zero bins, equally spaced from 0 with a width defined in the bin width parameter
- 𝑝(𝑖) be the normalized first order histogram and equal to 𝐏(𝑖)/𝑁𝑝

**1) Energy**


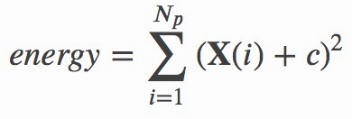


Energy is a measure of the magnitude of voxel values in an image. A larger value implies a greater sum of the squares of these values. 𝑐 is optional value, defined by voxelArrayShift, which shifts the intensities to prevent negative values in 𝐗. Because we are using CT data, we set this voxelArrayShift parameter to a fixed value 1000. In the IBSI feature definitions, no correction for negative gray values is implemented.

**2) Skewness**

The definition is in compliance with IBSI.

**3.2 Second-order features**

***A. Gray Level Size Zone Matrix (GLSZM) Features***

A Gray Level Size Zone (GLSZM) quantifies gray level zones in an image. A gray level zone is defined as the number of connected voxels that share the same gray level intensity. A voxel is considered connected if the distance is 1 according to the infinity norm (26-connected region in a 3D, 8-connected region in 2D). In a gray level size zone matrix 𝑃(𝑖,𝑗) the (𝑖,𝑗)^th^ element equals the number of zones with gray level 𝑖 and size 𝑗 appear in image. Contrary to GLCM and GLRLM, the GLSZM is rotation independent, with only one matrix calculated for all directions in the ROI [12]. As a two-dimensional example, consider the following 5x5 image, with 5 discrete gray levels:


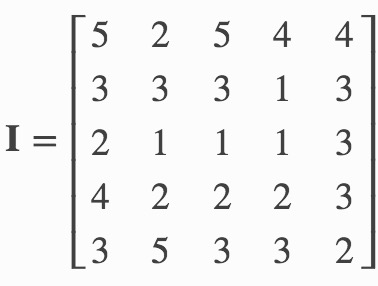


The GLSZM then becomes:


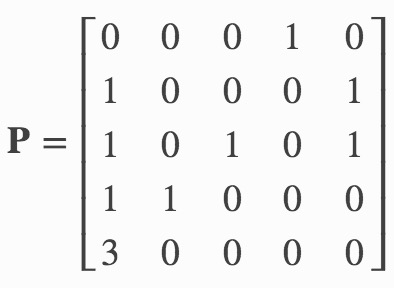


Let:

- 𝑁𝑔 be the number of discreet intensity values in the image
- 𝑁𝑠 be the number of discreet zone sizes in the image
- 𝑁𝑝 be the number of voxels in the image
- 𝑁𝑧 be the number of zones in the ROI, which is equal to
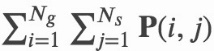
 and 1≤𝑁𝑧≤𝑁𝑝
- **𝐏**(𝑖,𝑗) be the size zone matrix
- 𝑝(𝑖,𝑗) be the normalized size zone matrix, defined as 
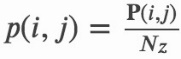


**1) Zone Variance (ZV)**

The definition is in compliance with IBSI.

**2) Large Area High Gray Level Emphasis (LAHGLE)**

The definition is in compliance with IBSI.

***B. Gray Level Run Length Matrix (GLRLM) Features***

A Gray Level Run Length Matrix (GLRLM) quantifies gray level runs, which are defined as the length in number of pixels, of consecutive pixels that have the same gray level value [13-16]. In a gray level run length matrix **𝐏**(𝑖,𝑗|𝜃), the (𝑖,𝑗)^th^ element describes the number of runs with gray level 𝑖 and length 𝑗 occur in the image (ROI) along angle 𝜃.

As a two-dimensional example, consider the following 5x5 image, with 5 discrete gray levels:


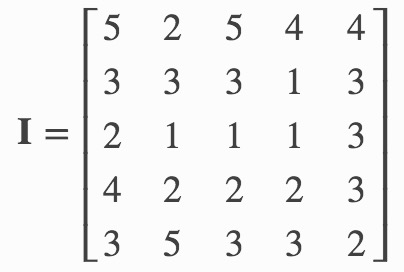


The GLRLM for 𝜃=0, where 0 degrees is the horizontal direction, then becomes:


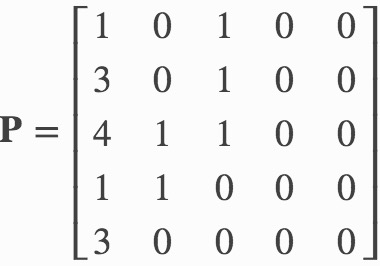


Let:

- 𝑁𝑔 be the number of discreet intensity values in the image
- 𝑁𝑟 be the number of discreet run lengths in the image
- 𝑁𝑝 be the number of voxels in the image
- 𝑁𝑟(𝜃) be the number of runs in the image along angle 𝜃, which is equal to
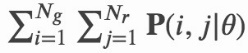
 and 1≤𝑁𝑟(𝜃)≤𝑁𝑝
- 𝐏(𝑖,𝑗|𝜃) be the run length matrix for an arbitrary direction 𝜃
- 𝑝(𝑖,𝑗|𝜃) be the normalized run length matrix, defined as 
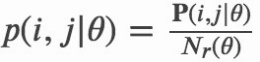


By default, the value of a feature is calculated on the GLRLM for each angle separately, after which the mean of these values is returned. If distance weighting is enabled, GLRLMs are weighted by the distance between neighboring voxels and then summed and normalized. Features are then calculated on the resultant matrix. The distance between neighboring voxels is calculated for each angle using the norm specified in ‘weightingNorm’.

**1) Long Run Emphasis (LRE)**

The definition is in compliance with IBSI.

**2) Low Gray Level Run Emphasis (LGLRE)**

The definition is in compliance with IBSI.

***C. Gray Level Dependence Matrix (GLDM) Features***

A Gray Level Dependence Matrix (GLDM) quantifies gray level dependencies in an image.[17] A gray level dependency is defined as the number of connected voxels within distance 𝛿 that are dependent on the center voxel. A neighboring voxel with gray level 𝑗j is considered dependent on center voxel with gray level 𝑖 if |𝑖−𝑗|≤𝛼. In a gray level dependence matrix **𝐏**(𝑖,𝑗) the (𝑖,𝑗)^th^ element describes the number of times a voxel with gray level 𝑖 with 𝑗 dependent voxels in its neighborhood appears in image. As a two-dimensional example, consider the following 5x5 image, with 5 discrete gray levels:


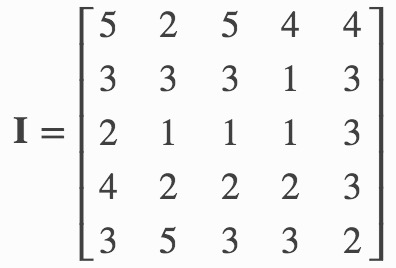


For 𝛼=0 and 𝛿=1, the GLDM then becomes:


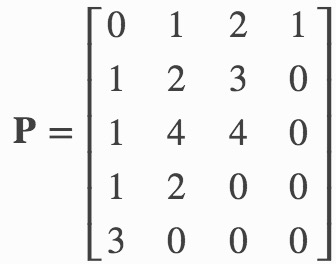


Let:

- 𝑁𝑔 be the number of discreet intensity values in the image
- 𝑁𝑑 be the number of discreet dependency sizes in the image
- 𝑁𝑧 be the number of dependency zones in the image, which is equal to 
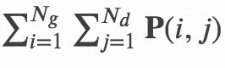

- 𝐏(𝑖,𝑗) be the dependence matrix
- 𝑝(𝑖,𝑗) be the normalized dependence matrix, defined as 
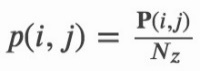


**Large Dependence Emphasis (LDE)**

The definition is in compliance with IBSI.

# Comparison of results in a combination of feature selection methods and classifiers

Different feature selection approaches were applied to the training data set including least absolute shrinkage and selection operator regression (LASSO), Elastic Net regression, recursive feature addition (RFA), and univariate feature selection by F-score. We examined five representative classification machine learning algorithms most commonly used in the context of radiomics studies. Logistic Regression (LR), Support Vector Machine (SVM), Random Forest (RF), XGboost Classifier (XG) and Decision tree (DT). Prediction performance of radiomics models was further assessed by the area under the receiver operating characteristic curve (AUC). The optimal combination was used as the standard method in the main manuscript.

# Statistical analysis

Radiomics features were harmonized to reduce the multicenter effect caused by different scanner and protocol settings. According to the statistical distribution of the dataset, nonparametric form of the model was adopted in which ComBat determined the transformation for each feature separately using “sva” R package [18]. Feature robustness was tested by intraclass correlation coefficients (ICCs) using “irr” R package [19]. Discrimination ability was assessed by Harrell's concordance indices (C-index) using “Hmisc” R package [20]. The 95% confidence intervals (CIs) for AUC accuracy, sensitivity, specificity, PPV and negative NPV were calculated based on confusion matrix with the use of “pROC” [14] ,“caret” [15] and “DTComPair” [16] R package.

# Supplementary Figures and Tables

**Table S1** Univariate clinical analysis on risk factors associated with LNM of patients in all patients

| Characteristics | LNM(-) (167) | LNM(+) (356) | P value |
| --- | --- | --- | --- |
| Age (mean ± SD, years) | 59.98 ± 10.53 | 59.47 ± 10.12 | 0.598 |
| Sex |  |  |  |
| Female | 44 | 90 | 0.878 |
| Male | 123 | 266 |  |
| Location |  |  |  |
| Cardia/fundus | 93 | 182 | 0.303 |
| Body | 34 | 77 |  |
| Antrum | 39 | 86 |  |
| More than two-thirds of stomach | 1 | 11 |  |
| Tumor thickness ± SD (mm) | 22.38 ± 8.35 | 22.97 ± 7.65 | 0.429 |
| Tumor diameter ± SD (mm) | 79.14 ± 39.09 | 93.04 ± 51.66 | 0.002* |
| Clinical T stage |  |  |  |
| T2 | 22 | 34 | <0.001* |
| T3 | 114 | 187 |  |
| T4a | 31 | 135 |  |
| CT-reported LN |  |  |  |
| Negative | 129 | 103 | <0.001* |
| Positive | 38 | 253 |  |

LNM: lymph node metastasis; (-): negative; (+): positive; *p < 0.05.

**Table S2** Predictive performance of radiological or clinical models in the training cohort

|  | AUC | Accuracy | Sensitivity | Specificity | PPV | NPV |
| --- | --- | --- | --- | --- | --- | --- |
| InceptionResNetV2 | 0.853 | 80.0 | 79.6 | 80.8 | 89.4 | 66.0 |
|  | (0.806, 0.911) | (72.3, 86.3) | (67.4, 84.2) | (75.2, 88.9) | (81.7, 93.7) | (53.7, 71.7) |
| VGG16 | 0.804 | 71.6 | 78.0 | 65.6 | 68.4 | 75.7 |
|  | (0.742, 0.906) | (63.2, 82.4) | 65.6, 86.9) | (60.3 72.8) | (60.7, 79.3) | (67.3, 88.4) |
| VGG19 | 0. 836 | 84.1 | 89.0 | 75.0 | 87.1 | 78.3 |
|  | (0.794，0.,895) | (78.1, 90.7) | (83.9, 95.2) | (67.2, 84.7) | (75.0, 93.9) | (69.7, 85.3) |
| ResNet50 | 0.897 | 82.5 | 78.7 | 90.5 | 94.7 | 66.5 |
|  | (0.836, 0.945) | (75.3, 87.6) | (69.4, 84.2) | (85.3, 94.1) | (88.3, 97.7) | (55.8, 75.7) |
| Xception | 0.842 | 78.0 | 72.9 | 88.3 | 92.7 | 61.5 |
|  | (0.806, 0.905) | (70.3, 85.1) | (67.4, 84.2) | (81.2, 93.9) | (83.7, 95.7) | (53.7, 74.7) |
| Radiomics | 0.779 | 74.0 | 77.5 | 66.4 | 83.2 | 57.9 |
|  | (0.712, 0.832) | (69.5, 83.5) | (70.8, 83.7) | (61.3, 75.6) | (75.3,89.1) | (48.9, 65.3) |
| Clinical signature | 0.756 | 73.0 | 70.0 | 78.9 | 86.7 | 57.1 |
|  | (0.701, 0.811) | (64.1, 78.3) | (65.0, 78.3) | (66.3, 82.6) | (75.6, 89.6) | (52.3, 65.5) |

Data are presented as percentages except AUC; 95% confidence intervals are included in parentheses. Abbreviations: AUC, area under the receiver operating characteristic curve; PPV, positive predictive value; NPV, negative predictive value.

**Table S3** Handcrafted radiomics features selected for the prediction models

| Index | Filter^a^ | Feature class | Feature |
| --- | --- | --- | --- |
| Radiomics (one slice with the maximum tumor lesion) | | | |
| 1 | Original^d^ | First order^c^ | Minimum |
| 2 | Original | Shape | Elongation |
| 3 | Original | Shape | Major Axis Length |
| 4 | Wavelet^b^ (HHH) | GLCM | Cluster Shade |
| 5 | Wavelet (HHH) | GLCM | Cluster Tendency |
| 6 | Wavelet (HHH) | GLDM | Dependence NonUniformity Normalized |
| 7 | Wavelet (HHH) | GLRLM | Run Variance |
| 8 | Wavelet (HHH) | GLSZM | Gray Level NonUniformity Normalized |
| 9 | Wavelet (HHH) | GLSZM | Large Area High Gray Level Emphasis |
| 10 | Wavelet (HHH) | GLSZM | Size Zone NonUniformity |
| 11 | Wavelet (HHH) | GLSZM | Small Area Low Gray Level Emphasis |
| 12 | Wavelet (HHH) | NGTDM | Strength |
| 13 | Wavelet (HHL) | First order | Range |
| 14 | Wavelet (HHL) | GLCM | Cluster Shade |
| 15 | Wavelet (HHL) | GLCM | Imc1 |
| 16 | Wavelet (HHL) | GLSZM | Zone Entropy |
| 17 | Wavelet (HHL) | GLSZM | Zone Variance |
| 18 | Wavelet (HLH) | First order | Kurtosis |
| 19 | Wavelet (HLH) | First order | Median |
| 20 | Wavelet (HLH) | GLCM | Maximum Probability |
| 21 | Wavelet (HLH) | GLDM | Dependence NonUniformity Normalized |
| 22 | Wavelet (HLH) | GLRLM | Run Variance |
| 23 | Wavelet (HLH) | GLRLM | Zone Percentage |
| 24 | Wavelet (HLL) | GLDM | Small Dependence High Gray Level Emphasis |
| 25 | Wavelet (HLL) | GLSZM | Gray Level NonUniformity Normalized |
| 26 | Wavelet (HLL) | GLSZM | Zone Entropy |
| 27 | Wavelet (LHH) | First order | Kurtosis |
| 28 | Wavelet (LHH) | First order | Median |
| 29 | Wavelet (LHH) | GLCM | Cluster Shade |
| 30 | Wavelet (LHH) | GLDM | Dependence Variance |
| 31 | Wavelet (LHH) | GLSZM | Large Area High Gray Level Emphasis |
| 32 | Wavelet (LHL) | First order | Median |
| 33 | Wavelet (LHL) | GLDM | Dependence NonUniformity Normalized |
| 34 | Wavelet (LHL) | NGTDM | Busyness |
| 35 | Wavelet (LLH) | GLCM | Cluster Shade |
| 36 | Wavelet (LLH) | GLCM | Correlation |
| 37 | Wavelet (LLH) | GLDM | Dependence NonUniformity Normalized |
| 38 | Wavelet (LLH) | GLDM | Small Dependence High Gray Level Emphasis |
| 39 | Wavelet (LLH) | GLDM | Small Dependence Low Gray Level Emphasis |
| 40 | Wavelet (LLH) | GLRLM | Long Run Low Gray Level Emphasis |
| 41 | Wavelet (LLH) | GLRLM | Short Run High Gray Level Emphasis |
| 42 | Wavelet (LLH) | GLSZM | Gray Level NonUniformity Normalized |
| 43 | Wavelet (LLH) | GLSZM | Large Area Emphasis |
| 44 | Wavelet (LLH) | GLSZM | Zone Variance |
| 45 | Wavelet (LLL) | GLCM | Imc |
| 46 | Wavelet (LLL) | GLSZM | Small Area Emphasis |
| 47 | Wavelet (LLL) | GLSZM | Zone Variance |
| 48 | Wavelet (LLL) | NGTDM | Contrast |

Abbreviations: HC, handcrafted radiomics; GLRLM, Gray Level Run Length Matrix Features; NGTDM, Neighbouring Gray Tone Difference Matrix; GLSZM, Gray Level Size Zone Matrix Features; GLDM, Gray Level Dependence Matrix Features; GLCM, Gray Level Co-occurrence Matrix Features; ^a^LLL, LHH, LLH and HHH represent high pass filter and low pass filter on the X, Y, Z three dimensions (H, high pass filter; L, low pass filter); ^b^Wavelet, wavelet filtrated image; ^c^First order, first order statistics; ^d^Original, original images without any filter applied.

**Table S4** Number of features used in DL-SVM models

| Feature extractor | Number of selected features |
| --- | --- |
| Xception | 63 |
| VGG16 | 56 |
| VGG19 | 79 |
| ResNet50 | 116 |
| InceptionResNetV2 | 90 |

Abbreviation: DL, deep learning.

**Table S5** Predictive performances of the DL-SVM models constructed from features extracted from different layers of ResNet

| **Layer** | **Training cohort** | | | | | | **Testing cohort** | | | | | |
| --- | --- | --- | --- | --- | --- | --- | --- | --- | --- | --- | --- | --- |
|  | **AUC** | **Accuracy** | **Sensitivity** | **Specificity** | **PPV** | **NPV** | **AUC** | **Accuracy** | **Sensitivity** | **Specificity** | **PPV** | **NPV** |
| Resnet50 | 0.897 | 82.5 | 78.7 | 90.5 | 94.7 | 66.5 | 0. 796 | 75.2 | 80.2 | 64.7 | 82.5 | 61.1 |
|  | (0.836, 0.945) | (75.3, 87.6) | (69.4, 84.2) | (85.3, 94.1) | (88.3, 97.7) | (55.8, 75.7) | (0.715-0.865) | (67.2, 81.5) | (75.4, 84.2) | (58.2, 71.6) | (74.9, 87.3) | (55.5, 69.3) |
| ResNet_res2b | 0.760 | 68.5 | 54.4 | 83.3 | 77.1 | 63.7 | 0..615 | 64.9 | 50.8 | 73.6 | 67.8 | 58.4 |
|  | (0.616, 0.830) | (60.4, 75.5) | (50.5, 63.0) | (75.0, 87.6) | (72.1, 85.2) | (59.6, 71.8) | (0.528, 0.691) | (53.3, 68.6) | (47.7, 61.4) | (65.0, 81.4) | (61.5, 75.5) | (52.1, 66.6) |
| ResNet_res3d | 0.891 | 88.5 | 89.5 | 86.4 | 93.2 | 79.7 | 0.582 | 56.1 | 56.5 | 55.1 | 73.5 | 36.5 |
|  | (0.842, 0.934) | (79.8, 92.7) | (85.4, 93.2) | (78.3, 89.1) | (87.6, 95.1) | (69.6, 83.7) | (0.526, 0.656) | (50.9, 63.8) | (50.1, 67.9) | (45.0, 60.1) | (66.0, 77.1) | (30.6, 41.9) |
| ResNet_res4f | 0.868 | 84.4 | 85.8 | 81.4 | 90.6 | 73.3 | 0.719 | 65.0 | 61.1 | 73.5 | 83.5 | 46.2 |
|  | (0.819, 0.939) | (81.2, 90.7) | (78.3, 92.2) | (71.2, 89.2) | (83.9, 95.7) | (69.3.5, 81.3) | (0.656, 0.753) | (60.6, 76.3) | (50.9, 66.8) | (66.7, 81.9) | (78.5, 87.1) | (42.6, 57.1) |
| ResNet_FC | 0.732 | 67.9 | 55.7 | 80.5 | 74.8 | 63.6 | 0.597 | 59.4 | 38.3 | 78.6 | 62.2 | 58.2 |
|  | (0.626, 0.781) | (55.1, 74.1) | (49.9, 65.7) | (72.5, 86.8) | (67.9, 80.5) | (57.7, 70.4) | (0.498, 0.675) | (48.9, 66.3) | (31.6, 45.9) | (70.9, 85.5) | (58.4, 69.6) | (51.3, 67.2) |

Data are presented as percentages except AUC; 95% confidence intervals are included in parentheses. Abbreviations: DL, deep learning; FC: Full connection; AUC, area under the receiver operating characteristic curve; PPV, positive predictive value; NPV, negative predictive value.

**Table S6** AUC of models built on multiple radiological features for the prediction of lymph node

| Feature selection methods | Classifiers | Feature extractor | Training cohort | Testing cohort |
| --- | --- | --- | --- | --- |
| LASSO | LR | Radiomics | 0.80419 | 0.69237 |
| LASSO | SVM | Radiomics | 0.90404 | 0.68552 |
| LASSO | RF | Radiomics | 0.89966 | 0.66190 |
| LASSO | XG | Radiomics | 0.85961 | 0.68472 |
| LASSO | DT | Radiomics | 0.85123 | 0.56597 |
| ElasticNet | LR | Radiomics | 0.81331 | 0.66984 |
| ElasticNet | SVM | Radiomics | 0.84608 | 0.69643 |
| ElasticNet | RF | Radiomics | 0.82986 | 0.67659 |
| ElasticNet | XG | Radiomics | 0.83123 | 0.68849 |
| ElasticNet | DT | Radiomics | 0.80345 | 0.53700 |
| RFA | LR | Radiomics | 0.57386 | 0.67667 |
| RFA | SVM | Radiomics | 0.76201 | 0.61667 |
| RFA | RF | Radiomics | 0.89614 | 0.60952 |
| RFA | XG | Radiomics | 0.89136 | 0.63194 |
| RFA | DT | Radiomics | 0.80236 | 0.47034 |
| Univariate+LASSO | LR | Radiomics | 0.7359 | 0.66926 |
| Univariate+LASSO | SVM | Radiomics | 0.87156 | 0.70407 |
| Univariate+LASSO | RF | Radiomics | 0.89952 | 0.69994 |
| Univariate+LASSO | XG | Radiomics | 0.89979 | 0.70325 |
| Univariate+LASSO | DT | Radiomics | 0.89523 | 0.58176 |
| Univariate+ElasticNet | LR | Radiomics | 0.73591 | 0.66926 |
| Univariate+ElasticNet | SVM | Radiomics | 0.86856 | 0.60007 |
| Univariate+ElasticNet | RF | Radiomics | 0.89952 | 0.71994 |
| Univariate+ElasticNet | XG | Radiomics | 0.89979 | 0.71569 |
| Univariate+ElasticNet | DT | Radiomics | 0.90021 | 0.58176 |
| Univariate+RFA | LR | Radiomics | 0.54946 | 0.57048 |
| Univariate+RFA | SVM | Radiomics | 0.82723 | 0.67518 |
| Univariate+RFA | RF | Radiomics | 0.88806 | 0.69108 |
| Univariate+RFA | XG | Radiomics | 0.87881 | 0.71032 |
| Univariate+RFA | DT | Radiomics | 0.79235 | 0.54477 |
| LASSO | LR | InceptionResNetV2 | 0.54683 | 0.66778 |
| LASSO | SVM | InceptionResNetV2 | 0.66499 | 0.59083 |
| LASSO | RF | InceptionResNetV2 | 0.87632 | 0.63300 |
| LASSO | XG | InceptionResNetV2 | 0.84239 | 0.66149 |
| LASSO | DT | InceptionResNetV2 | 0.79565 | 0.47799 |
| ElasticNet | LR | InceptionResNetV2 | 0.54683 | 0.66778 |
| ElasticNet | SVM | InceptionResNetV2 | 0.76499 | 0.59083 |
| ElasticNet | RF | InceptionResNetV2 | 0.87632 | 0.63300 |
| ElasticNet | XG | InceptionResNetV2 | 0.84239 | 0.66149 |
| ElasticNet | DT | InceptionResNetV2 | 0.90125 | 0.47799 |
| RFA | LR | InceptionResNetV2 | 0.55131 | 0.47857 |
| RFA | SVM | InceptionResNetV2 | 0.77118 | 0.52510 |
| RFA | RF | InceptionResNetV2 | 0.89919 | 0.53373 |
| RFA | XG | InceptionResNetV2 | 0.89128 | 0.58889 |
| RFA | DT | InceptionResNetV2 | 0.89578 | 0.47470 |
| Univariate+LASSO | LR | InceptionResNetV2 | 0.79243 | 0.69326 |
| Univariate+LASSO | SVM | InceptionResNetV2 | 0.88615 | 0.70767 |
| Univariate+LASSO | RF | InceptionResNetV2 | 0.89973 | 0.60692 |
| Univariate+LASSO | XG | InceptionResNetV2 | 0.90125 | 0.61968 |
| Univariate+LASSO | DT | InceptionResNetV2 | 0.91323 | 0.59813 |
| Univariate+ElasticNet | LR | InceptionResNetV2 | 0.86874 | 0.63908 |
| Univariate+ElasticNet | SVM | InceptionResNetV2 | 0.89307 | 0.66493 |
| Univariate+ElasticNet | RF | InceptionResNetV2 | 0.90125 | 0.61654 |
| Univariate+ElasticNet | XG | InceptionResNetV2 | 0.86254 | 0.59545 |
| Univariate+ElasticNet | DT | InceptionResNetV2 | 0.89523 | 0.53533 |
| Univariate+RFA | LR | InceptionResNetV2 | 0.54306 | 0.58571 |
| Univariate+RFA | SVM | InceptionResNetV2 | 0.80418 | 0.65033 |
| Univariate+RFA | RF | InceptionResNetV2 | 0.89687 | 0.58690 |
| Univariate+RFA | XG | InceptionResNetV2 | 0.88815 | 0.63472 |
| Univariate+RFA | DT | InceptionResNetV2 | 0.88257 | 0.62143 |
| LASSO | LR | Resnet50 | 0.54815 | 0.65483 |
| LASSO | SVM | Resnet50 | 0.76037 | 0.55808 |
| LASSO | RF | Resnet50 | 0.85005 | 0.61043 |
| LASSO | XG | Resnet50 | 0.73931 | 0.58472 |
| LASSO | DT | Resnet50 | 0.85612 | 0.57436 |
| ElasticNet | LR | Resnet50 | 0.77672 | 0.71214 |
| ElasticNet | SVM | Resnet50 | 0.86031 | 0.72323 |
| ElasticNet | RF | Resnet50 | 0.86979 | 0.66500 |
| ElasticNet | XG | Resnet50 | 0.85979 | 0.68572 |
| ElasticNet | DT | Resnet50 | 0.90677 | 0.53061 |
| RFA | LR | Resnet50 | 0.60554 | 0.48372 |
| RFA | SVM | Resnet50 | 0.86653 | 0.51905 |
| RFA | RF | Resnet50 | 0.94922 | 0.59452 |
| RFA | XG | Resnet50 | 0.94702 | 0.56067 |
| RFA | DT | Resnet50 | 0.86023 | 0.52590 |
| Univariate+LASSO | LR | Resnet50 | 0.86557 | 0.72863 |
| Univariate+LASSO | SVM | Resnet50 | 0.89764 | 0.79597 |
| Univariate+LASSO | RF | Resnet50 | 0.88998 | 0.64576 |
| Univariate+LASSO | XG | Resnet50 | 0.86871 | 0.64132 |
| Univariate+LASSO | DT | Resnet50 | 0.85361 | 0.53931 |
| Univariate+ElasticNet | LR | Resnet50 | 0.81224 | 0.70847 |
| Univariate+ElasticNet | SVM | Resnet50 | 0.86999 | 0.76101 |
| Univariate+ElasticNet | RF | Resnet50 | 0.87997 | 0.61191 |
| Univariate+ElasticNet | XG | Resnet50 | 0.87354 | 0.61062 |
| Univariate+ElasticNet | DT | Resnet50 | 0.86258 | 0.52987 |
| Univariate+RFA | LR | Resnet50 | 0.77524 | 0.65483 |
| Univariate+RFA | SVM | Resnet50 | 0.81622 | 0.56770 |
| Univariate+RFA | RF | Resnet50 | 0.85934 | 0.62190 |
| Univariate+RFA | XG | Resnet50 | 0.89433 | 0.60969 |
| Univariate+RFA | DT | Resnet50 | 0.80235 | 0.58833 |
| LASSO | LR | VGG16 | 0.58496 | 0.53849 |
| LASSO | SVM | VGG16 | 0.66973 | 0.48423 |
| LASSO | RF | VGG16 | 0.89124 | 0.53413 |
| LASSO | XG | VGG16 | 0.86227 | 0.52679 |
| LASSO | DT | VGG16 | 0.83634 | 0.53929 |
| ElasticNet | LR | VGG16 | 0.62313 | 0.53452 |
| ElasticNet | SVM | VGG16 | 0.74665 | 0.49127 |
| ElasticNet | RF | VGG16 | 0.89927 | 0.51687 |
| ElasticNet | XG | VGG16 | 0.89941 | 0.47500 |
| ElasticNet | DT | VGG16 | 0.84536 | 0.50139 |
| RFA | LR | VGG16 | 0.56338 | 0.45218 |
| RFA | SVM | VGG16 | 0.72137 | 0.49603 |
| RFA | RF | VGG16 | 0.89827 | 0.48710 |
| RFA | XG | VGG16 | 0.89621 | 0.50238 |
| RFA | DT | VGG16 | 0.83952 | 0.47679 |
| Univariate+LASSO | LR | VGG16 | 0.61131 | 0.59742 |
| Univariate+LASSO | SVM | VGG16 | 0.70445 | 0.66125 |
| Univariate+LASSO | RF | VGG16 | 0.8982 | 0.60536 |
| Univariate+LASSO | XG | VGG16 | 0.89729 | 0.59944 |
| Univariate+LASSO | DT | VGG16 | 0.90123 | 0.52361 |
| Univariate+ElasticNet | LR | VGG16 | 0.6373 | 0.53929 |
| Univariate+ElasticNet | SVM | VGG16 | 0.7751 | 0.58657 |
| Univariate+ElasticNet | RF | VGG16 | 0.89841 | 0.57103 |
| Univariate+ElasticNet | XG | VGG16 | 0.8998 | 0.58397 |
| Univariate+ElasticNet | DT | VGG16 | 0.93752 | 0.52798 |
| Univariate+RFA | LR | VGG16 | 0.53266 | 0.50933 |
| Univariate+RFA | SVM | VGG16 | 0.63166 | 0.56161 |
| Univariate+RFA | RF | VGG16 | 0.88886 | 0.52143 |
| Univariate+RFA | XG | VGG16 | 0.85167 | 0.52738 |
| Univariate+RFA | DT | VGG16 | 0.8063 | 0.58135 |
| LASSO | LR | VGG19 | 0.65988 | 0.61746 |
| LASSO | SVM | VGG19 | 0.74358 | 0.59260 |
| LASSO | RF | VGG19 | 0.89702 | 0.59970 |
| LASSO | XG | VGG19 | 0.89738 | 0.55401 |
| LASSO | DT | VGG19 | 0.80236 | 0.46744 |
| ElasticNet | LR | VGG19 | 0.61764 | 0.61723 |
| ElasticNet | SVM | VGG19 | 0.70076 | 0.58161 |
| ElasticNet | RF | VGG19 | 0.89639 | 0.59896 |
| ElasticNet | XG | VGG19 | 0.89336 | 0.56215 |
| ElasticNet | DT | VGG19 | 0.83624 | 0.55855 |
| RFA | LR | VGG19 | 0.54584 | 0.54828 |
| RFA | SVM | VGG19 | 0.68418 | 0.57529 |
| RFA | RF | VGG19 | 0.89384 | 0.60026 |
| RFA | XG | VGG19 | 0.88726 | 0.56900 |
| RFA | DT | VGG19 | 0.75236 | 0.52405 |
| Univariate+LASSO | LR | VGG19 | 0.63802 | 0.61553 |
| Univariate+LASSO | SVM | VGG19 | 0.80382 | 0.57827 |
| Univariate+LASSO | RF | VGG19 | 0.8999 | 0.640798 |
| Univariate+LASSO | XG | VGG19 | 0.89997 | 0.54347 |
| Univariate+LASSO | DT | VGG19 | 0.90125 | 0.53931 |
| Univariate+ElasticNet | LR | VGG19 | 0.6392 | 0.62222 |
| Univariate+ElasticNet | SVM | VGG19 | 0.79638 | 0.60016 |
| Univariate+ElasticNet | RF | VGG19 | 0.89986 | 0.58861 |
| Univariate+ElasticNet | XG | VGG19 | 0.90123 | 0.53496 |
| Univariate+ElasticNet | DT | VGG19 | 0.80625 | 0.46670 |
| Univariate+RFA | LR | VGG19 | 0.53485 | 0.60895 |
| Univariate+RFA | SVM | VGG19 | 0.7477 | 0.53182 |
| Univariate+RFA | RF | VGG19 | 0.85439 | 0.64835 |
| Univariate+RFA | XG | VGG19 | 0.88601 | 0.64909 |
| Univariate+RFA | DT | VGG19 | 0.75456 | 0.51970 |
| LASSO | LR | Xception | 0.61954 | 0.62956 |
| LASSO | SVM | Xception | 0.77749 | 0.60635 |
| LASSO | RF | Xception | 0.88918 | 0.59444 |
| LASSO | XG | Xception | 0.87787 | 0.58294 |
| LASSO | DT | Xception | 0.82523 | 0.57708 |
| ElasticNet | LR | Xception | 0.5921 | 0.65476 |
| ElasticNet | SVM | Xception | 0.78462 | 0.55079 |
| ElasticNet | RF | Xception | 0.88801 | 0.57440 |
| ElasticNet | XG | Xception | 0.88032 | 0.54286 |
| ElasticNet | DT | Xception | 0.81347 | 0.50575 |
| RFA | LR | Xception | 0.51811 | 0.57778 |
| RFA | SVM | Xception | 0.75814 | 0.58393 |
| RFA | RF | Xception | 0.85727 | 0.59325 |
| RFA | XG | Xception | 0.87287 | 0.57520 |
| RFA | DT | Xception | 0.75125 | 0.52351 |
| Univariate+LASSO | LR | Xception | 0.74865 | 0.65316 |
| Univariate+LASSO | SVM | Xception | 0.87145 | 0.66024 |
| Univariate+LASSO | RF | Xception | 0.84053 | 0.64558 |
| Univariate+LASSO | XG | Xception | 0.85292 | 0.62653 |
| Univariate+LASSO | DT | Xception | 0.82231 | 0.53496 |
| Univariate+ElasticNet | LR | Xception | 0.82654 | 0.63836 |
| Univariate+ElasticNet | SVM | Xception | 0.88515 | 0.65111 |
| Univariate+ElasticNet | RF | Xception | 0.89975 | 0.64278 |
| Univariate+ElasticNet | XG | Xception | 0.84725 | 0.62227 |
| Univariate+ElasticNet | DT | Xception | 0.78215 | 0.46633 |
| Univariate+RFA | LR | Xception | 0.56142 | 0.57024 |
| Univariate+RFA | SVM | Xception | 0.72999 | 0.58379 |
| Univariate+RFA | RF | Xception | 0.86948 | 0.54802 |
| Univariate+RFA | XG | Xception | 0.88012 | 0.52798 |
| Univariate+RFA | DT | Xception | 0.8352 | 0.51687 |

Abbreviations: AUC, area under the receiver operating characteristic curve. Feature selection methods: LASSO, least absolute shrinkage and selection operator regression; Elastic Net regression; RFA, recursive feature addition; univariate feature selection by F-score. Classifiers: LR, linear regression; SVM, support vector machine; RF, random forest; XG, XGboost; DT, decision tree

**Figure S1** Comparison of the mean ranks of AUC of models built by using cross combination of different feature selection methods and classifiers


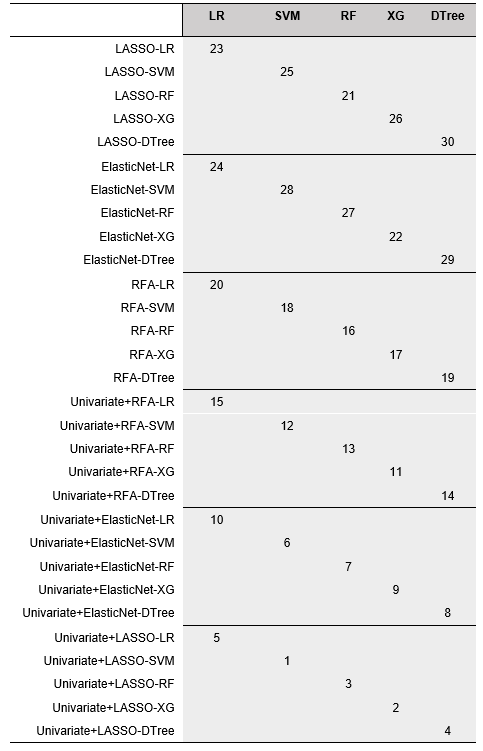


**Figure S2** Evaluation of predictive performance of the combined model


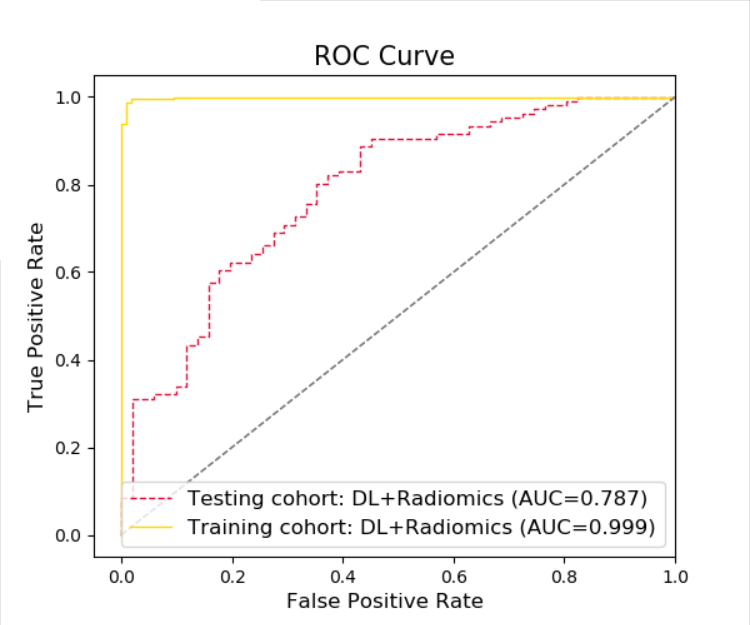


The receiver operating characteristic curve shows the predictive power of the model incorporating deep learning (DL) and handcrafted radiomics features.

**Figure S3** Evaluation of predictive performance of the combined model (clinical + DL)


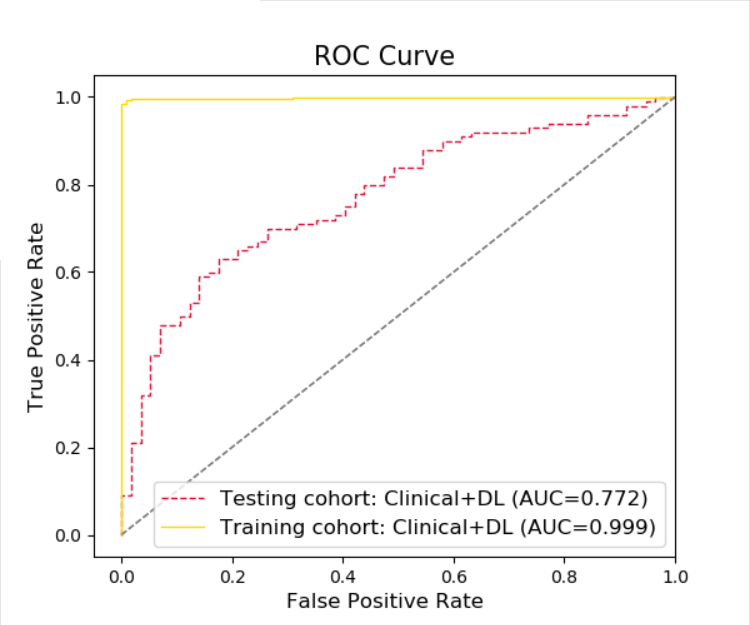


The receiver operating characteristic curve shows the predictive power of the model incorporating clinical and deep learning (DL) features.

**Figure S4** Evaluation of predictive performance of the combined model (clinical + Radiomics)


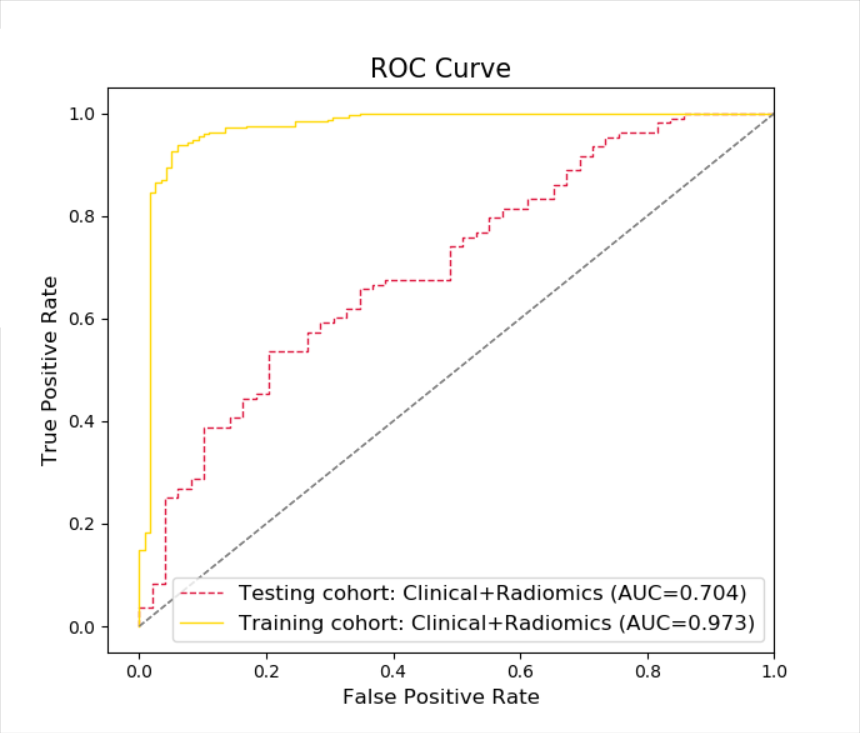


The receiver operating characteristic curve shows the predictive power of the model incorporating clinical and handcrafted radiomics features.

**References:**

1. Li J, Dong D, Fang M, et al. Dual-energy CT-based deep learning radiomics can improve lymph node metastasis risk prediction for gastric cancer. [European](http://www.baidu.com/link?url=XJW7qte6Bi4iaA5MPEzMzPeFZXm8r5NdsrSKxVctZMl4mK-lt7CO6Ir8_R28LycLJi9zYiyEgIlJ3U64g0A4Mq) Radiology, 2020. 30: p. 2324-2333.
2. Silva AC, Morse BG, Hara AK, Paden RG, Hongo N, Pavlicek W. Dual-energy (spectral) CT: applications in abdominal imaging. RadioGraphics, 2011. 31:1031-1046: p. 1047-1050.
3. Zhao, H.P., et al. TCGA-TCIA–Based CT Radiomics Study for Noninvasively Predicting Epstein-Barr Virus Status in Gastric Cancer. [American Journal of Roentgenology](https://www.ajronline.org/loi/ajr) , 2021. 217(1): p. 124-134.
4. Simonyan, K. and A. Zisserman, Very Deep Convolutional Networks for Large-Scale Image Recognition. CoRR, 2014. abs/1409.1556.
5. Szegedy, C., et al., Rethinking the Inception Architecture for Computer Vision. 2016 IEEE Conference on Computer Vision and Pattern Recognition (CVPR), 2015: p. 2818-2826.
6. He, K., et al., Deep Residual Learning for Image Recognition. 2016 IEEE Conference on Computer Vision and Pattern Recognition (CVPR), 2016: p. 770-778.
7. Szegedy, C., et al. Inception-v4, Inception-ResNet and the Impact of Residual Connections on Learning. in AAAI. 2016.
8. Chollet, F., Xception: Deep Learning with Depthwise Separable Convolutions. 2017 IEEE Conference on Computer Vision and Pattern Recognition (CVPR), 2016: p. 1800-1807.
9. Russakovsky, O., et al., Imagenet large scale visual recognition challenge. International journal of computer vision, 2015. 115(3): p. 211-252.
10. Van Griethuysen, J.J., et al., Computational radiomics system to decode the radiographic phenotype. Cancer Res, 2017. 77(21): p. e104-e107.
11. Zwanenburg, A., et al., Image biomarker standardisation initiative. J arXiv preprint arXiv:.07003, 2016.
12. Thibault, G., et al., Shape and texture indexes application to cell nuclei classification. International Journal of Pattern Recognition Artificial Intelligence, 2013. 27(01): p. 1357002.
13. Galloway, M., Texture classification using gray level run length. Computer graphics image processing, 1975. 4(2): p. 172-179.
14. Chu, A., C.M. Sehgal, and J.F. Greenleaf, Use of gray value distribution of run lengths for texture analysis. Pattern Recognition Letters, 1990. 11(6): p. 415-419.
15. Tang, X., Texture information in run-length matrices. IEEE transactions on image processing, 1998. 7(11): p. 1602-1609.
16. Xu, D.-H., et al., Run-length encoding for volumetric texture, in The 4th IASTED International Conference on Visualization, Imaging and Image Processing – VIP. 2004: Marbella, Spain. p. 452-458.
17. Sun, C. and W.G. Wee, Neighboring gray level dependence matrix for texture classification. Computer Vision, Graphics, and Image Processing, 1983. 23(3): p. 341-352.
18. Leek, J.T., et al., The sva package for removing batch effects and other unwanted variation in high-throughput experiments. Bioinformatics, 2012. 28(6): p. 882-3.
19. Gamer, M., et al., Package irr: Various Coefficients of Interrater Reliability and Agreement (versión 0.84). 2012.
20. Harrell Jr, F.E. and M.F.E. Harrell Jr, Package ‘Hmisc’. J CRAN, 2019: p. 235-6.
